# MiR‐23a‐3p alleviates cigarette smoke extract‐induced pulmonary vascular endothelial cell apoptosis by targeting DNAJB1 in emphysema

**DOI:** 10.1111/crj.13707

**Published:** 2023-10-12

**Authors:** Ke Li, Xianwei Ye, Mei Xu, Chuan Xu, Ping Lu, Jiayi Li, Guohang Yuan, Cheng Zhang

**Affiliations:** ^1^ Department of Respiratory and Critical Care Medicine Guizhou Provincial People's Hospital Guiyang City People's Republic of China; ^2^ Department of Thoracic Surgery Guizhou Provincial People's Hospital Guiyang City People's Republic of China

**Keywords:** cigarette smoke, DNAJB1, emphysema, miR‐23a‐3p

## Abstract

**Background:**

Cigarette smoke (CS) is an important risk factor for chronic obstructive pulmonary disease, including emphysema. MicroRNAs (miRNAs) are important regulators of emphysema progression. However, miR‐23a‐3p role in emphysema is unclear.

**Methods:**

CS exposure was used to construct emphysema mice models, and cigarette smoke extract (CSE)‐induced pulmonary vascular endothelial cells (PMVECs) were used to mimic emphysema cell models. Mouse lung tissue was stained by immunohistochemical staining, hematoxylin and eosin staining, and TUNEL staining. MiR‐23a‐3p and DnaJ homolog subfamily B member 1 (DNAJB1) levels were tested using quantitative real‐time PCR. DNAJB1 and apoptosis‐related markers' protein levels were examined via western blot analysis. Cell viability and apoptosis were analyzed by MTT assay and flow cytometry. The interaction between miR‐23a‐3p and DNAJB1 was evaluated by dual‐luciferase reporter assay and RIP assay.

**Results:**

MiR‐23a‐3p was downregulated, and DNAJB1 was upregulated in CS‐induced emphysema mice models and CSE‐induced PMVECs. MiR‐23a‐3p overexpression promoted viability and repressed apoptosis in CSE‐induced PMVECs. MiR‐23a‐3p targeted DNAJB1 and negatively regulated DNAJB1 expression. Moreover, DNAJB1 knockdown repressed CSE‐induced PMVECs apoptosis, and miR‐23a‐3p inhibitor reversed this effect. Additionally, miR‐23a‐3p alleviated lung tissue injury and improved emphysema in mice by reducing DNAJB1 expression.

**Conclusion:**

MiR‐23a‐3p alleviated emphysema progression, which could inhibit CSE‐induced PMVECs apoptosis by targeting DNAJB1.

## Graphical abstract

The present study revealed that miR‐23a‐3p repressed cigarette smoke‐induced emphysema in mice and CSE‐induced PMVECs apoptosis by targeting DNAJB1.

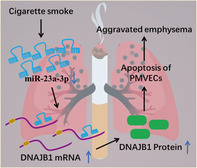

## 1 INTRODUCTION

Emphysema, a common form of chronic obstructive pulmonary disease (COPD), is characterized by lung parenchyma destruction.^1^, ^2^ Cigarette smoke (CS) is an indoor air pollutant with strong oxidative, pro‐inflammatory, and carcinogenic properties and is a major risk factor for emphysema.^3^, ^4^ Pulmonary vascular endothelial cells (PMVECs) are single‐layer flat cells located in the inner surface of pulmonary circulation vessels, which participate in regulating various pathological and physiological processes.^5^, ^6^ Recent research have indicated that apoptosis of PMVECs is closely related to emphysema development.^7^, ^8^ Therefore, elucidating the molecular mechanism affecting PMVECs apoptosis induced by cigarette smoke extract (CSE) is expected to provide new ideas for treating emphysema.

MicroRNAs (miRNAs) are non‐coding RNAs with a length of about 20–23 nts that regulate cellular functions by binding to target genes.^9^, ^10^ Many miRNAs have been shown to mediate the progression of COPD, including emphysema.^11^ For example, miR‐221‐3p might be a target for COPD treatment, which was lowly expressed in COPD patients, and its overexpression repressed CSE‐induced cell inflammation and apoptosis.^12^ Through GEO database screening and further analysis, Liu et al. found that miR‐23a had decreased expression in COPD patients and might be a promising biomarker for distinguishing between frequent and infrequent exacerbations in COPD patients.^13^ However, miR‐23a roles in emphysema progression have not been studied.

DnaJ homolog subfamily B member 1 (DNAJB1, Hsp40 member B1), a member of the heat shock protein family, regulates cellular processes by aiding in the folding, transport, and assembly.^14^, ^15^ Studies have shown that DNAJA1 may be an effective target for treating radiation resistance in many cancers.^16^ By the screening of GEO database, Sun et al. pointed out that DNAJB1 expression was increased in COPD patients, suggesting that DNAJB1 might be a potential regulatory factor for COPD development.^17^ However, the specific role of DNAJB1 in emphysema is not yet known.

Here, we discovered that miR‐23a‐3p and DNAJB1 were differentially expressed in CS‐induced emphysema mice models and CSE‐induced PMVECs, and bioinformatics analysis indicated that there were potential binding sites between them. Therefore, we proposed the hypothesis that miR‐23a‐3p regulated emphysema progression through DNAJB1, hoping to provide potential molecular targets for emphysema treatment.

## 2 METHODS

### 2.1 Emphysema mice models

C57BL/6J mice (6 weeks old, Vital River, Beijing, China) were randomly divided into four groups (*n* = 4/group). As previously described,^18^ mice exposed to normal air were the control group, and those exposed to CS with total particulate matter (300 mg/m^3^, 2 h/day) using the Baumgartner–Jaeger CS machine were the CS group. Besides, mice were administered with adenovirus miR‐23a‐3p mimics or NC mimics (Genechem, Shanghai, China) by intranasal instillation and then exposed to CS, which was the CS + miR‐23a‐3p mimics group or CS + NC mimics group. After 16 weeks of exposure, lung tissue samples were collected for analysis and testing. Animal studies were approved by the Ethics Committee of Guizhou Provincial People's Hospital and in compliance with the Guide for the Care and Use of Laboratory Animals.

### 2.2 Immunohistochemical (IHC), hematoxylin and eosin (H&E), and TUNEL staining

Lung tissue samples of mice were exposed to 10% formalin and subsequently dehydrated and paraffin‐embedded to prepare into 4‐μm sections. For IHC staining, lung tissue section was incubated with anti‐DNAJB1 (1:50, ab231577, Abcam, Cambridge, MA, USA) and secondary antibodies after dewaxing, inactivation, and sealing using SP Kit (Solarbio, Beijing, China). Then, the sections were treated with DAB solution and hematoxylin followed by detected DNAJB1 positive cells under a microscope. For H&E staining, section was hatched with hematoxylin solution, Bluing Reagent, and Eosin Y Solution using H&E Staining Kit (Abcam). After being washed with absolute alcohol, the pathological changes of mice lung tissue section were observed under a microscope. For TUNEL staining, section was dewaxed, inactivated, and sealed, followed by hatched with Biotin labeling solution Streptavidin‐HRP solution, DAB solution, and hematoxylin using TUNEL Apoptosis Assay Kit (Beyotime, Shanghai, China). Then, the apoptosis of mice lung tissue cells was counted under a microscope.

### 2.3 CSE preparation

The smoke from the 3R4F Research Cigarettes (University of Kentucky, Lexington, Kent, USA) was sucked through a vacuum pump at a constant rate into a flask containing culture medium, and the collected suspension was titrated to pH 7.4 and sterilized through a 0.2‐mm pore filter to prepare CSE solution. CSE was diluted to prepare 0.25% CSE solution for cell treatment.

### 2.4 PMVECs isolation, culture, and treatment

According to the previously described,^8^ C57BL/6J mice were anesthetized, and then the lung tissue was collected and enzymolized using Mouse Lung Dissociation Kit (Miltenyi Biotec, Bergisch Gladbach, Germany). CD45− cells were collected and incubated with CD31‐conjugated beads (Invitrogen, Carlsbad, CA, USA) to enrich CD31+ cells by MACS columns and magnetic fields. Newly isolated PMVECs suspended in PMVECs complete culture medium were cultured in a dish coated with human fibronectin at 37°C with 5% CO_2_. PMVECs were exposed with 0.25% CSE solution for 12 h to mimic emphysema cell models as previously described.^8^ PMVECs were transfected with miR‐23a‐3p mimics or inhibitors, DNAJB1 short hairpin RNA (sh‐DNAJB1), and their controls (NC mimics, NC inhibitor, and sh‐NC) with Lipofectamine 3000 (Invitrogen) before CSE exposure.

### 2.5 Quantitative real‐time PCR (qRT‐PCR)

RNA was isolated by TRIzol reagent (Invitrogen) and its concentrations were assessed by NanoDrop spectrophotometer. PrimeScript RT reagent kit (Takara, Dalian, China) was used to obtain cDNA, and qRT‐PCR was performed by mixing of cDNA, specific primers (Table 1), and SYBR Green (Takara). Data were calculated by 2^−ΔΔCt^ method with U6 (for miR‐23a‐3p) or β‐actin (for DNAJB1) as housekeeping gene.

**TABLE 1:** Primer sequences used for qRT‐PCR.

Gene	Forward (5′‐3′)	Reverse (5′‐3′)
miR‐23a‐3p	ATCACATTGCCAGGGATTTCC	GAACATGTCTGCGTATCTC
U6	CTCGCTTCGGCAGCACATATACT	ACGCTTCACGAATTTGCGTGTC
DNAJB1	TTCGACCGCTATGGAGAGGAA	CACCGAAGAACTCAGCAAACA
β‐actin	GGACTGTTACTGAGCTGCGTT	CGCCTTCACCGTTCCAGTT

### 2.6 Western blot (WB) analysis

Total proteins were extracted from PMVECs or lung tissues using RIPA lysis buffer (Beyotime). After being loaded on 10% SDS‐PAGE gel, protein was transferred onto PVDF membranes. Membranes were treated with anti‐DNAJB1 (ab231577, Abcam), anti‐Bax (ab32503, Abcam), anti‐cleaved‐caspase3 (ab2302, Abcam), anti‐Bcl‐2 (ab32124, Abcam), and anti‐β‐actin (ab8227, Abcam) with 1:1000 dilution, followed by incubated with secondary antibody (ab205718, Abcam). Then, ECL reagent (Beyotime) was employed to develop the protein bands.

### 2.7 Cell viability detection

Basing on the instructions of MTT Kit (Solarbio), PMVECs seeded into 96‐well plates were cultured overnight. Subsequently, cells were hatched with 0.5% MTT solution and treated with Formazan dissolving solution. Cell viability was measured at 490 nm by a microplate reader.

### 2.8 Cell apoptosis detection

PMVECs were collected and suspended with binding buffer followed by incubated with Annexin V‐FITC and PI solution followed by instructions of the Annexin V‐FITC Apoptosis Detection Kit (Beyotime). Cell apoptosis was analyzed under FACSCalibur flow cytometry and CELLQUEST program.

### 2.9 Dual‐luciferase reporter assay

The sequences of DNAJB1 3'UTR containing the binding sites or mutant sites of miR‐23a‐3p were sub‐cloned into the pmirGLO vectors and generated the wild‐type or mutant‐type DNAJB1 (WT/MUT‐DNAJB1) vector. WT/MUT‐DNAJB1 vectors were transfected into PMVECs with miR‐23a‐3p mimics or inhibitors. Relative luciferase activity was examined using Dual‐Luciferase Reporter Assay System Kit (Promega, Madison, WI, USA).

### 2.10 RIP assay

Using Magna RIP Kit (Millipore, Bedford, MA, USA), the lysates of PMVECs transfected with miR‐23a‐3p mimics/NC mimics were hatched with magnetic beads‐coupled with Ago2 antibody or IgG antibody. DNAJB1 mRNA expression in immunoprecipitated RNA was analyzed using qRT‐PCR.

### 2.11 Statistical analysis

Data are shown as mean ± SD and analyzed with GraphPad 7.0 software. Unpaired Student's *t*‐test was used for comparison between two groups. ANOVA test was used for comparisons between three or more groups, and Tukey's multiple comparisons test was used for post hoc comparisons. We have added this information in revised manuscript.

Three replicates were used in cell experiment, and four replicates were used in animal experiment. Statistical significance was set at *P* < 0.05.

## 3 RESULTS

### 3.1 miR‐23a‐3p was downregulated and DNAJB1 was upregulated in emphysema mice models and cell models

**FIGURE 1:**
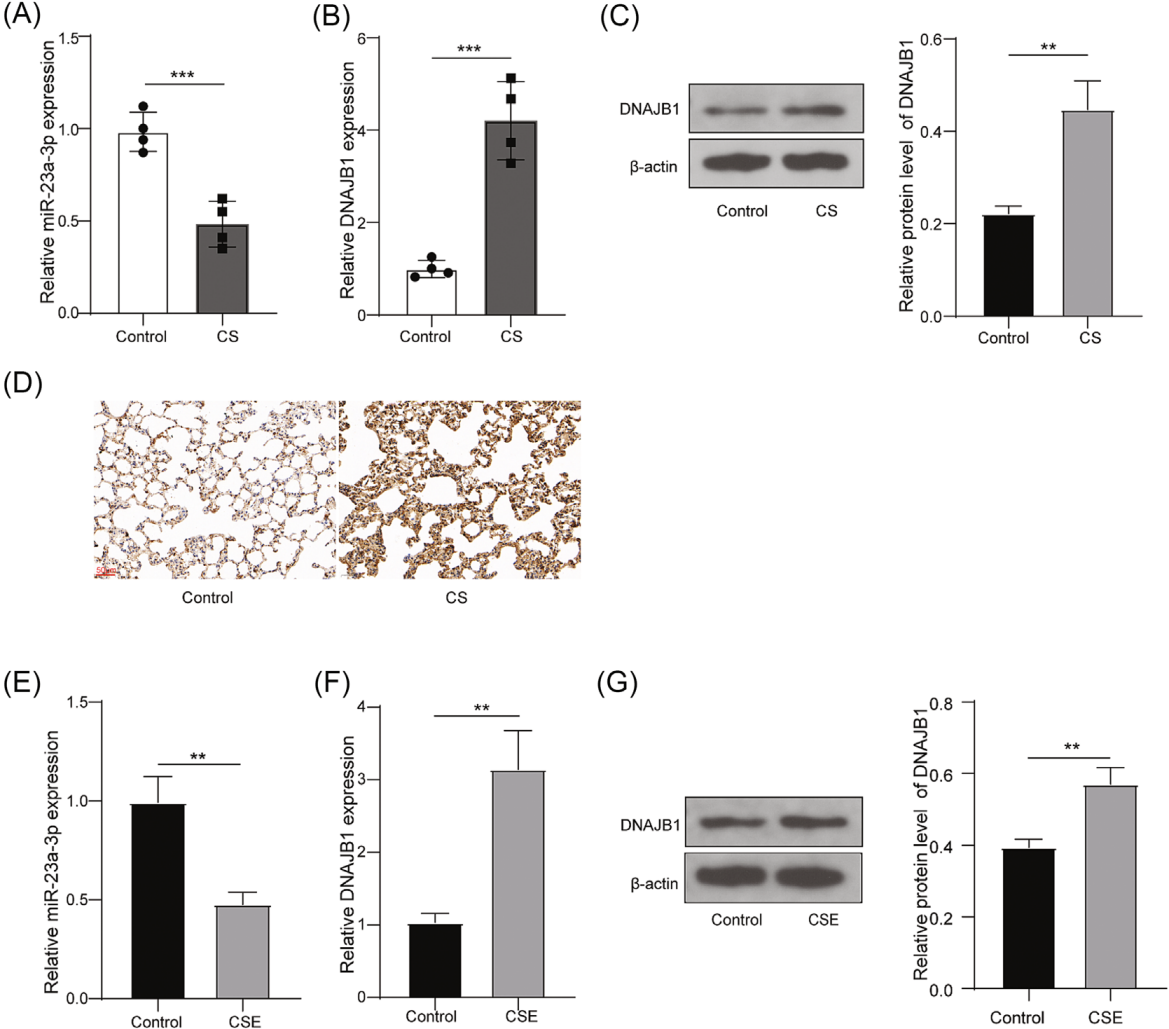
miR‐23a‐3p and DNAJB1 expression in emphysema mice models and cell models. (A–B) miR‐23a‐3p and DNAJB1 mRNA expression in the lung tissues of CS‐induced emphysema mice models and control mice was examined by qRT‐PCR. (C) DNAJB1 protein expression was tested using WB analysis in the lung tissues of CS‐induced emphysema mice models and control mice. (D) DNAJB1 expression in the lung tissues of CS‐induced emphysema mice models and control mice was examined by IHC staining. (E) miR‐23a‐3p expression in PMVECs exposed with or without CSE was examined by qRT‐PCR. (F–G) DNAJB1 mRNA and protein expression in PMVECs exposed with or without CSE was evaluated by qRT‐PCR and WB analysis, respectively. **, *P* < 0.01; ***, *P* < 0.001.

In the lung tissues of CS‐induced emphysema mice models, miR‐23a‐3p was lowly expressed, whereas DNAJB1 was highly expressed (Figure 1A–B). WB analysis confirmed the high expression of DNAJB1, and IHC staining further detected the increased DNAJB1 expression in the lung tissues of CS‐induced emphysema mice models (Figure 1C–D). In addition, CSE‐induced PMVECs were used to construct emphysema cell models. MiR‐23a‐3p had decreased expression, whereas DNAJB1 had elevated expression at the mRNA and protein levels in CSE‐induced PMVECs (Figure 1E–G). The above data illuminated that miR‐23a‐3p and DNAJB1 were dysexpressed in emphysema models.

### 3.2 Overexpressed miR‐23a‐3p could inhibit CSE‐induced PMVECs apoptosis

**FIGURE 2:**
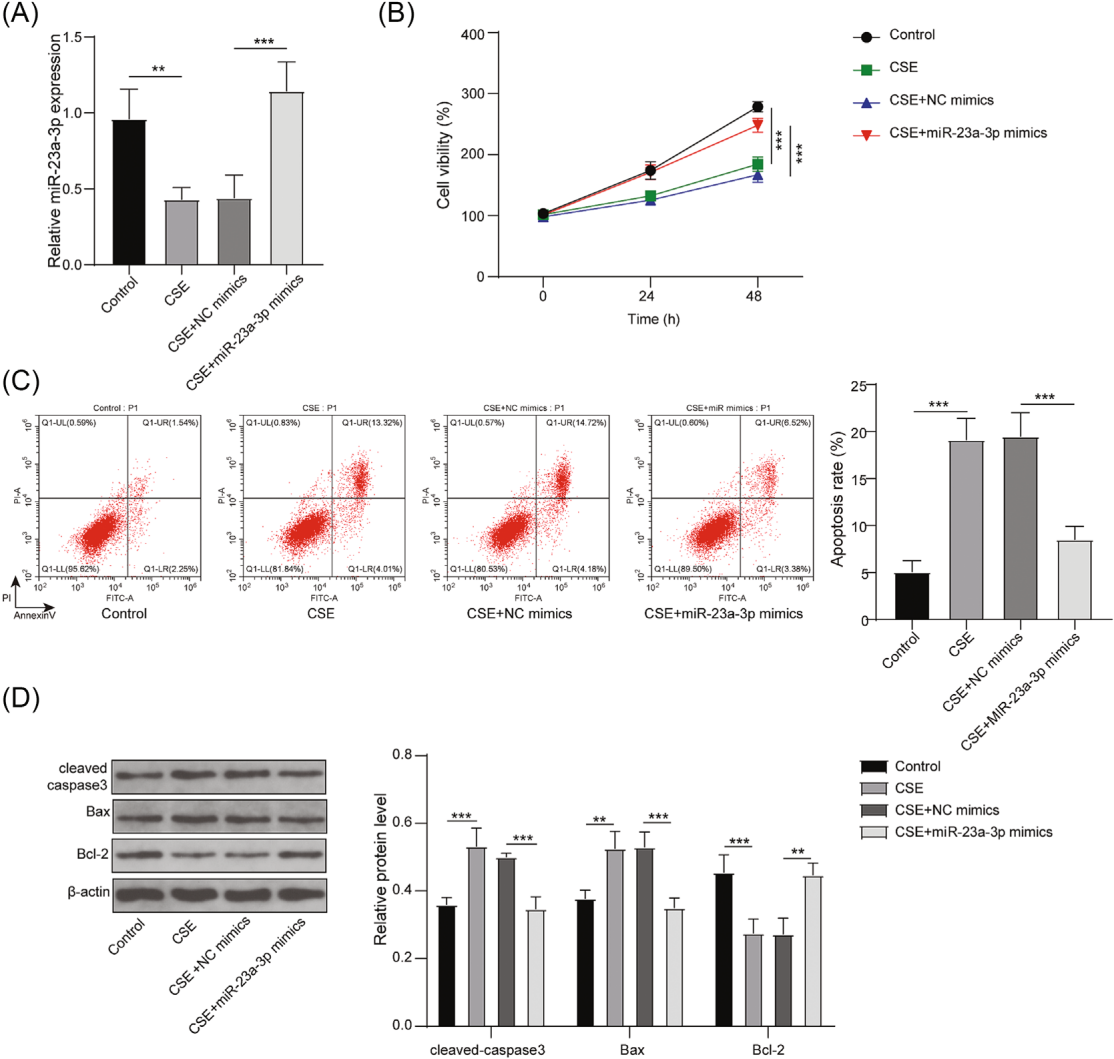
Effect of miR‐23a‐3p mimics on CSE‐induced PMVECs apoptosis. PMVECs were transfected with NC mimics/miR‐23a‐3p mimics and exposed to CSE. (A) miR‐23a‐3p expression was tested using qRT‐PCR. (B) Cell viability was examined using MTT assay. (C) Cell apoptosis was measured by flow cytometry. (D) Protein levels were detected by WB analysis. **, *P* < 0.01; ***, *P* < 0.001.

To explore miR‐23a‐3p roles in emphysema, PMVECs were transfected with miR‐23a‐3p mimics/NC mimics and then exposed to CSE. The transfection of miR‐23a‐3p mimics significantly enhanced miR‐23a‐3p level in CSE‐induced PMVECs (Figure 2A). Functionally, CSE treatment suppressed PMVECs viability and aggravated apoptosis, whereas miR‐23a‐3p overexpression enhanced viability and reduced apoptosis in CSE‐induced PMVECs (Figure 2B–C). Besides, CSE treatment increased cleaved‐caspase3 protein level and Bax protein level and decreased Bcl‐2 protein level, whereas these effects were abolished by miR‐23a‐3p overexpression (Figure 2D). These data showed that upregulation of miR‐23a‐3p restrained CSE‐induced PMVECs apoptosis, confirming that miR‐23a‐3p might restrain emphysema progression.

### 3.3 miR‐23a‐3p targeted DNAJB1

**FIGURE 3:**
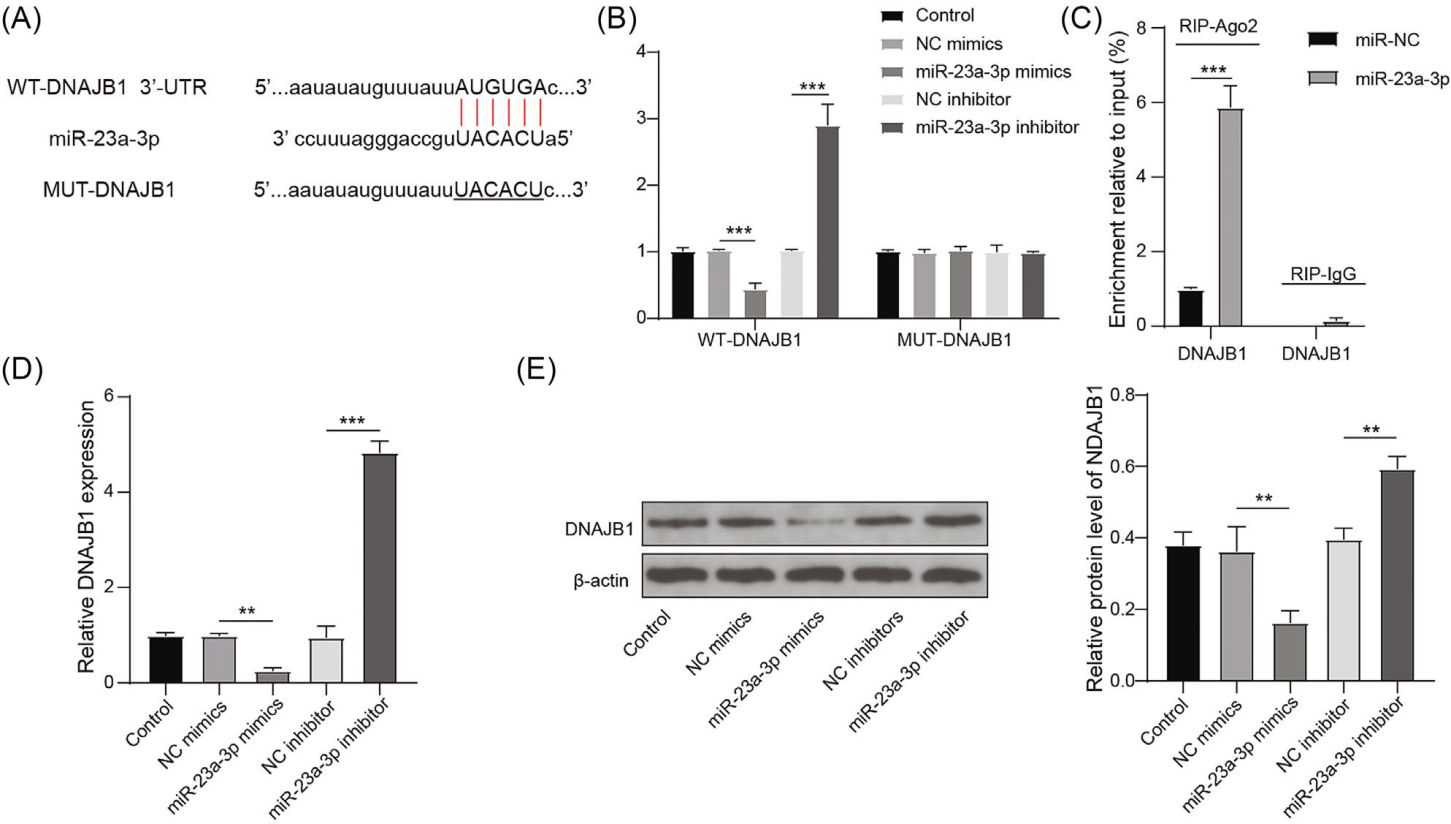
miR‐23a‐3p targeted DNAJB1. (A) miR‐23a‐3p and DNAJB1 binding sites are shown. Dual‐luciferase reporter assay (B) and RIP assay (C) were used to assess RNA interaction. (D–E) DNAJB1 mRNA and protein expression in PMVECs transfected with miR‐23a‐3p mimics or inhibitor was evaluated by qRT‐PCR and WB analysis. **, *P* < 0.01; ***, *P* < 0.001.

The starbase software predicated that miR‐23a‐3p could bind to DNAJB1 3'UTR (Figure 3A). Also, the luciferase activity of WT‐DNAJB1 vector could be suppressed by miR‐23a‐3p mimics and enhanced by miR‐23a‐3p inhibitor, but the luciferase activity of MUT‐DNAJB1 vector had not any changed (Figure 3B). In addition, RIP results showed that DNAJB1 was significantly enriched in Ago2 after overexpressing miR‐23a‐3p (Figure 3C). Moreover, DNAJB1 mRNA and protein expression levels were inhibited by miR‐23a‐3p mimics and promoted by its inhibitors (Figure 3D–E). The above data revealed that miR‐23a‐3p targeted DNAJB1.

### 3.4 miR‐23a‐3p regulated CSE‐induced PMVECs apoptosis by targeting DNAJB1

**FIGURE 4:**
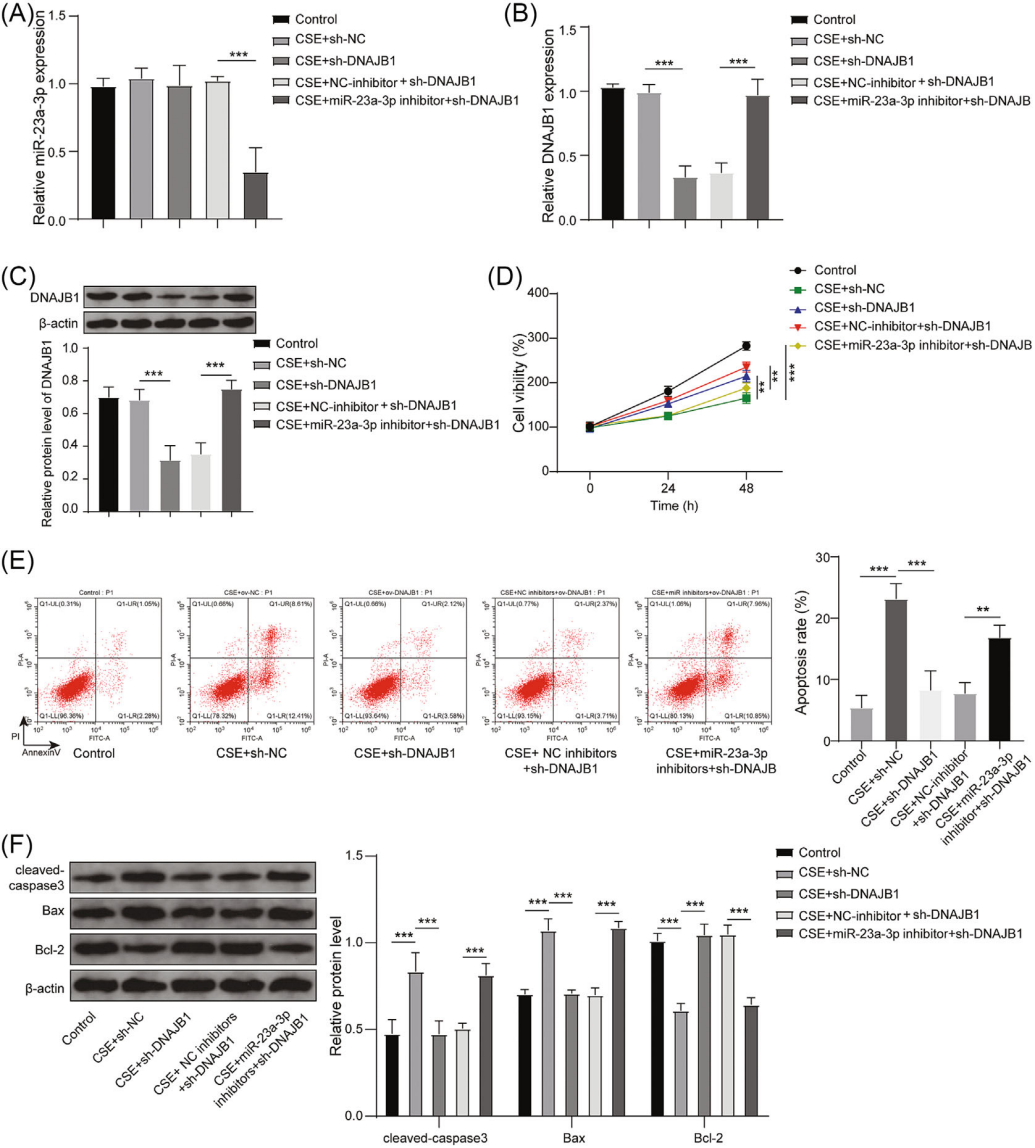
Effect of sh‐DNAJB1 and miR‐23a‐3p inhibitors on CSE‐induced PMVECs apoptosis. PMVECs were transfected with sh‐NC, sh‐DNAJB1, NC inhibitors+sh‐DNAJB1 or miR‐23a‐3p inhibitors+sh‐DNAJB1. (A) miR‐23a‐3p expression was tested using qRT‐PCR. (B–C) qRT‐PCR and WB analysis were employed to measure DNAJB1 mRNA and protein expression. (D–F) Transfected PMVECs were exposed to CSE. MTT assay (D) and flow cytometry (E) were performed to examine cell viability and apoptosis. (F) WB analysis was used to test protein levels. *, *P* < 0.05; **, *P* < 0.01; ***, *P* < 0.001.

To determine DNAJB1 roles in CSE‐induced PMVECs and confirm whether miR‐23a‐3p mediated emphysema progression by regulating DNAJB1, PMVECs were transfected with sh‐DNAJB1 and miR‐23a‐3p inhibitors. Detection results of miR‐23a‐3p expression suggested that miR‐23a‐3p inhibitors markedly reduced miR‐23a‐3p expression, whereas sh‐DNAJB1 had no effect on its expression (Figure 4A). Besides, sh‐DNAJB1 effectively decreased DNAJB1 mRNA and protein expression, whereas miR‐23a‐3p inhibitors eliminated this effect (Figure 4B–C). These data confirmed that DNAJB1 was the downstream gene of miR‐23a‐3p, and its expression was regulated by miR‐23a‐3p. DNAJB1 knockdown promoted viability and repressed apoptosis in CSE‐induced PMVECs, whereas miR‐23a‐3p inhibitors partially abolished sh‐DNAJB1‐mediated CSE‐induced PMVECs viability and apoptosis (Figure 4D–E). Also, DNAJB1 knockdown decreased cleaved‐caspase3 level, reduced Bax level, and enhanced Bcl‐2 level, whereas these effects were eliminated by miR‐23a‐3p inhibitors (Figure 4F). The above data indicated that silencing DNAJB1 played an anti‐apoptotic role in CSE‐induced PMVECs, and miR‐23a‐3p might regulate emphysema progression via targeting DNAJB1.

### 3.5 miR‐23a‐3p improved emphysema in mice through DNAJB1

**FIGURE 5:**
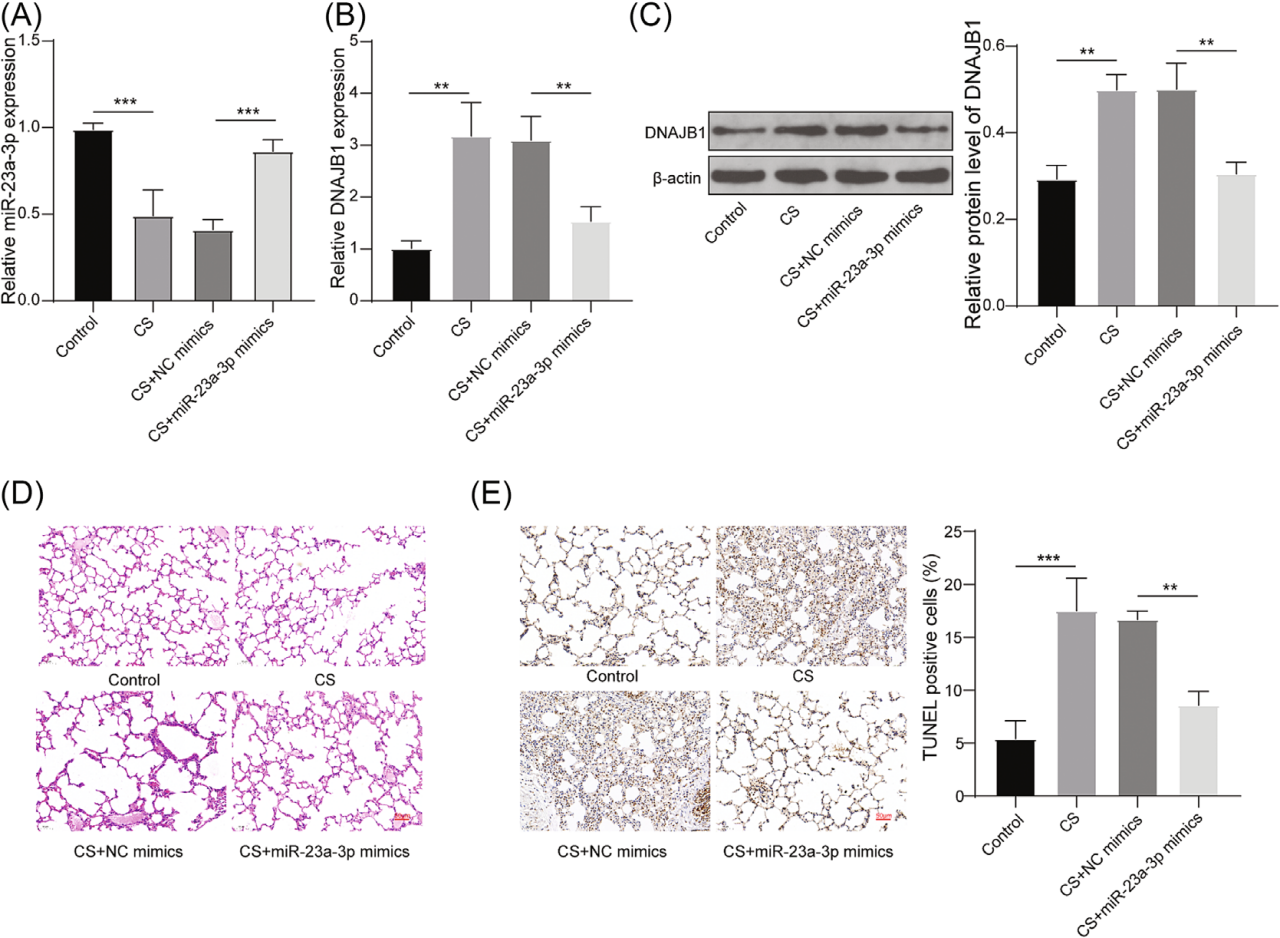
Effect of miR‐23a‐3p mimics on CS‐induced emphysema in mice. Mice were administered with adenovirus miR‐23a‐3p mimics or NC mimics and then exposed to CS. (A) miR‐23a‐3p expression in the lung tissues of mice was tested using qRT‐PCR. (B–C) DNAJB1 mRNA and protein expression in the lung tissues of mice was assessed using qRT‐PCR and WB analysis. (D) H&E staining was used to observe the pathological changes of mice lung tissues. (E) The apoptosis of lung tissue cells was detected by TUNEL staining. **, *P* < 0.01; ***, *P* < 0.001.

To further confirm that miR‐23a‐3p targeted DNAJB1 to mediate emphysema progression, in vivo study was performed. In the lung tissues of CS‐induced emphysema mice models, miR‐23a‐3p mimics significantly elevated miR‐23a‐3p expression and reduced DNAJB1 mRNA and protein expression (Figure 5A–C). H&E staining showed that CS exposure caused the destruction of alveolar wall and irregular expansion of the gas gap, and miR‐23a‐3p overexpression alleviated CS‐induced emphysema injury in mice (Figure 5D). In addition, TUNEL staining results revealed that TUNEL‐positive cells were reduced in miR‐23a‐3p mimics group (Figure 5E), confirming that miR‐23a‐3p overexpression reversed CS‐induced lung tissue cell apoptosis. These data revealed that miR‐23a‐3p alleviated CS‐induced emphysema progression in mice through DNAJB1.

## 4 DISCUSSION

As a common form of COPD, patients with emphysema often experience varying degrees of difficulty breathing, along with coughing, expectoration, loss of appetite, and general fatigue.^19^ Many data confirm that CS is an important risk factor for many lung diseases, including emphysema.^20^ In recent years, many studies have shown that patients with emphysema have an unbalanced balance of PMVECs proliferation and apoptosis, and PMVECs apoptosis may play a role in emphysema pathogenesis.^21^, ^22^ Therefore, our study explored the functions of miR‐23a‐3p and DNAJB1 in CS‐induced emphysema in mice and CSE‐induced PMVECs apoptosis, with a view to providing potential molecular targets for emphysema treatment.

MiR‐23a‐3p has been confirmed to refer to regulating various disease processes by mediating cell proliferation and apoptosis.^23^ MiR‐23a‐3p had increased expression in colon cancer tissues and could elevate cancer cell proliferation and inhibit apoptosis.^24^ In addition, mR‐23a‐3p might be a novel therapeutic target for sepsis‐associated acute kidney injury (AKI), which was low expressed in AKI patients and could elevate proliferation and repress apoptosis in LPS‐induced HK‐2 cells.^25^ Based on the analysis results of Liu et al.,^13^ we selected miR‐23a‐3p as the object and explored its role in the process of emphysema. In this, miR‐23a‐3p was indeed downregulated in a CS‐induced emphysema mice model, and its high expression alleviated CS‐induced lung tissue injury in mice. In CSE‐induced PMVECs, miR‐23a‐3p accelerated cell viability and hindered apoptosis. Besides, a previous study showed that miR‐23a‐3p overexpression reduced the protein levels of pro‐apoptosis markers (Bax and cleaved‐caspase3) to alleviate high glucose‐induced cardiomyocyte apoptosis.^26^ Consistent with this study, our data confirmed that miR‐23a‐3p could inhibit the protein levels of cleaved‐caspase3 and Bax and enhance the protein level of anti‐apoptosis marker (Bcl‐2) in CSE‐induced PMVECs, verifying that miR‐23a‐3p might a negative regulator of apoptotic signal proteins. These results support the conclusion that miR‐23a‐3p may be used to alleviate emphysema progression.

Further, we demonstrated that miR‐23a‐3p bound to DNAJB1 complementally. DNAJB1, located at 19p13.2, plays an important role in protein translation, folding/refolding, transport, and degradation.^27^ DNAJB1‐PRKACA fusion transcripts are considered to be oncogenic drivers of fibrolamellar hepatocellular carcinoma.^28^ Studies had shown that DNAJB1 was highly expressed and might be recognized as a self‐antigen in lung cancer patients.^29^ Here, we detected that DNAJB1 was overexpressed in the CS‐induced emphysema mice model, which was consistent with the high DNAJB1 level in COPD patients indicated by Sun et al.^17^ Functional experiments showed that DNAJB1 overexpression aggravated PMVECs apoptosis induced by CSE and could overturn miR‐23a‐3p‐mediated the inhibition on CSE‐induced PMVECs apoptosis. Also, miR‐23a‐3p negatively regulated DNAJB1 expression. The above data confirmed that miR‐23a‐3p targeted DNAJB1 to mediate emphysema progression.

## 5 CONCLUSIONS

To sum up, our data concluded that miR‐23a‐3p repressed CS‐induced emphysema in mice and CSE‐induced PMVECs apoptosis by targeting DNAJB1. Our findings revealed that targeted miR‐23a‐3p/DNAJB1 axis might be used for emphysema treatment, which provided a novel therapeutic strategy for emphysema.

## AUTHOR CONTRIBUTIONS

Ke Li and Cheng Zhang is the guarantor of the integrity of the entire study; Xianwei Ye contributed to the study concepts and study design; Mei Xu contributed to the definition of intellectual content; Chuan Xu contributed to the literature research; Ping Lu contributed to the experimental studies, data acquisition, and data analysis; Jiayi Li contributed to the statistical analysis; Guohang Yuan contributed to the manuscript preparation; Ke Li and Cheng Zhang contributed to manuscript editing and manuscript review.

## CONFLICT OF INTEREST STATEMENT

The authors declare that they have no conflict of interest.

## ETHICS STATEMENT

Animal studies were approved by the Ethics Committee of Guizhou Provincial People's Hospital and in compliance with the Guide for the Care and Use of Laboratory Animals.

## Data Availability

The datasets used or analyzed during this study can be made available from the corresponding author upon reasonable request.
